# Surface enolase is a virulence factor of *Leishmania amazonensis* metacyclic promastigotes involved in adhesion to macrophages

**DOI:** 10.3389/fmicb.2026.1789827

**Published:** 2026-06-10

**Authors:** Franciane dos Santos Alves, Lisvane Paes, Marta Teixeira Gomes, Suzana Passos Chaves, Marcello André Barcinski, José Roberto Meyer-Fernandes, David S. Ucker, João Luiz Mendes Wanderley

**Affiliations:** 1 Laboratório de Imunoparasitologia, Departamento de Biociências Aplicadas a Saúde, Instituto de Ciências Médicas, Universidade Federal do Rio de Janeiro, Macaé, Brazil; 2 Medical Biochemistry Institute, Federal University of Rio de Janeiro, Rio de Janeiro, Brazil; 3 Biophysics Institute, Federal University of Rio de Janeiro, Rio de Janeiro, Brazil; 4 Department of Microbiology and Immunology, College of Medicine, University of Illinois, Chicago, IL, United States

**Keywords:** cell surface, enolase, *Leishmania amazonensis*, *Leishmania* differentiation, macrophage adhesion, metacyclic promastigotes, parasite virulence factors

## Abstract

We find that enolase, an evolutionarily conserved and multifunctional protein, is displayed on the cell surface of *Leishmania amazonensis* parasites in a developmentally regulated manner, associated with infectivity. *Leishmania* surface enolase acts as a parasite virulence factor. Specifically, surface exposure of enolase is limited to the infective metacyclic promastigote form in viable parasites. Correspondingly, *L. amazonensis* surface enolase plays a role specifically in the adhesion of parasites to host macrophages, the target cell type for productive infection. Surface enolase also may be involved in triggering the immunologically quiescent host response associated with early *L. amazonensis* infection. The display of enolase on the *L. amazonensis* promastigote parallels its exposure on the surface of apoptotic cells of metazoans as well as on the surface of bacteria and other pathogens that appear to mimic apoptotic cells. As in the case of enolase displayed on the apoptotic cell surface, externalized *Leishmania* enolase lacks glycolytic enzymatic activity. Our data reinforce the view that surface enolase functions significantly in the interaction of viable host cells with apoptotic corpses and with pathogens that exploit apoptotic mimicry.

## Introduction

*Leshmania* species are significant intracellular protozoan pathogens. They are the etiological agents of leishmaniasis associated with a spectrum of pathologies ranging from cutaneous lesions to fatal visceral infections. *Leishmania amazonensis* is responsible for some of the most severe manifestations of the cutaneous disease, such as disseminated and diffuse leishmaniasis. Leshmaniasis is a poverty-related disease and is endemic in the developing world, particularly in South America, Africa, and southern Asia. It is estimated that 12 million individuals are infected worldwide, with as many as 1 million new cases occurring every year (WHO, 2023). The pathogen also has emerged as an important opportunistic infection associated with HIV (van Griensven et al., 2014).

During infection, the interface of microbial pathogens with host processes involving cell death is complex and occurs on multiple levels. Of particular note, apoptotic cells are cleared efficiently by phagocytic cells and, independently, elicit profound host immune suppression generally (Birge and Ucker, 2008; Cvetanovic and Ucker, 2004; Fadok et al., 1998; Savill et al., 2002; Voll et al., 1997). Subversion or mimicry of these aspects of apoptotic cell behaviors appear frequently to be approaches employed by pathogens to promote their infectivity or enhance their replication and spread (Ucker, 2016). For example, some pathogens trigger immunosuppression through the induction of apoptosis in host cells, as has been demonstrated for the bacterium *Listeria monocytogenes* (Pattabiraman et al., 2017) and another kinetoplastid *Trypanosoma cruzi* (Nunes et al., 1998). Other pathogens express surface molecules that mirror or substitute for authentic apoptotic ligands to ensure their engulfment into target cells (DaMatta et al., 2007; dos Santos et al., 2011; Mercer and Helenius, 2008; Wanderley et al., 2006). Still others express agonists that trigger host immune suppression and promote an environment permissive for the pathogen (van Zandbergen et al., 2006; Wanderley et al., 2013). Molecular dissection of this mimicry may be revealing of the essential elements of the respective host functions and their apoptotic modulation.

Parasites of the genus *Leishmania* alternate between a free-living, flagellated promastigote form in insect vectors and an intracellular, rounded amastigote form in infected mammalian hosts. Within the insect vector, replicating (procyclic) promastigotes further differentiate into a non-replicative (metacyclic) infectious form. The internalization of *Leishmania* parasites into mammalian macrophage hosts has been shown to occur in a manner dependent on the exposure of phosphatidylserine (Wanderley et al., 2006, 2009). Just as the anti-inflammatory milieu elicited by apoptotic cells enhances leishmanial growth (Afonso et al., 2008), *Leishmania* themselves promote the induction of anti-inflammatory cytokines in infected mammalian hosts (de Oliveira and Brodskyn, 2012). Among these, it is striking that TGF-β, in addition to suppressing host inflammatory responses, also serves as a stimulus for leishmanial growth (Barral et al., 1993). Especially under conditions of chronic *Leishmania* infection, systemic cytokine profiles in infected individuals resemble the apoptotic-like anti-inflammatory setting (de Oliveira and Brodskyn, 2012; Ji et al., 2003).

Enolase is a molecule of interest in this context. Enolase is an evolutionarily conserved and multifunctional protein, classically identified by virtue of its cytoplasmic role as an enzyme catalyzing the conversion of 2-phosphoglycerate to phosphoenolpyruvate within the glycolytic pathway. Enolase, especially the ubiquitously expressed α isoform, also exerts non-metabolic functions, both intra- and extra-cellularly (Agarwal et al., 2012; Decker and Wickner, 2006; Entelis et al., 2006; Fong-ngern and Thongboonkerd, 2016; Subramanian and Miller, 2000; Wang et al., 2005; Wistow et al., 1988), most notably as a plasminogen receptor (discussed below). In addition, enolase is displayed on the mammalian cell surface during the process of apoptotic cell death (Ucker et al., 2012). Surface enolase expression has been reported on oncogenically transformed cells as well, although apoptotic cells within the tumor cell population may account for many of these.

Enolase is also a cell-surface protein of a variety of pathogens. A suggested role for bacterial surface enolase, linked to its ability to bind and serve as an activation site for plasminogen, is in facilitating the vascular migration and spread of bacteria, especially by circumventing fibrin clots (Attali et al., 2008; Hussain et al., 2022; Zhao et al., 2024). Other pathogens, including *Leishmania*, expose enolase, and may thereby enhance their pathogenicity (Vanegas et al., 2007).

While the function of surface enolase on the apoptotic cell surface is unknown, it has been suggested to play a role in recognition leading to apoptotic cell clearance and/or the suppression of host immunity (Ucker et al., 2012). It seems unlikely that the property of dissemination is advantageous to apoptotic cells, although it may be important for tumor metastasis (Hagan et al., 2025). During the process of apoptosis, the surface exposure of mammalian enolase occurs coincidentally with the externalization of almost the entire set of enzyme molecules that comprise the glycolytic pathway, so called SUPER (surface-exposed, ubiquitously expressed, protease-sensitive, evolutionarily conserved, and already resident) determinants characteristic of the apoptotic cell surface (Ucker et al., 2012). As with enolase, it is unclear what roles those other glycolytic enzyme molecules might play on the cell surface.

Here, we describe results of our examination of the expression of surface enolase by different forms of *Leishmania amazonensis* and its associated function during macrophage infection.

## Results

### Enolase is exposed on the cell surface of viable metacyclic *L. amazonensis* promastigotes in a developmentally-restricted manner

We explored the surface exposure of enolase on *Leishmania amazonensis*. *L. amazonensis* parasites were cultured *in vitro* under conditions that allowed promastigote differentiation and the acquisition of infectivity. Parasites obtained on day 1 of fresh culture after at least 5 short-term consecutive passages (logarithmic-phase), and from 5- to 7-days cultures (stationary-phase), were stained with anti-enolase antibodies and assessed by flow cytometry. In particular, we employed an enolase-specific rabbit antiserum directed to recombinant enolase from *L. mexicana* (a related New World *Leishmania* species), kindly provided by Dr. Wilfredo Quiñones [Los Andes University; (Quiñones et al., 2007)]. (Hereafter, we denote this enolase-specific rabbit serum as EnoV).

**FIGURE 1:**
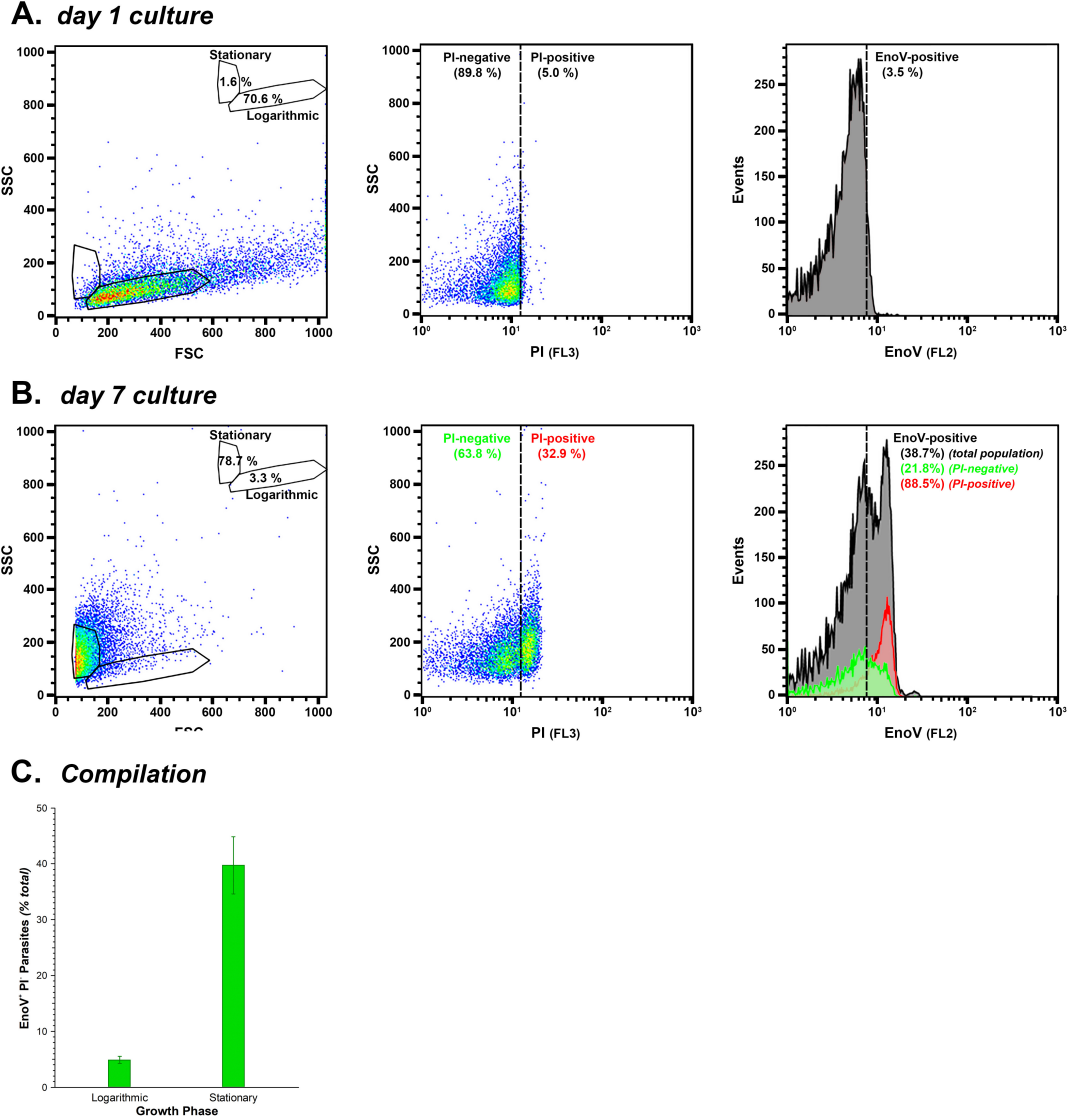
Characterization of surface enolase expression on stationary- and logarithmic-phase *L. amazonensis* parasites. *L. amazonensis* promastigotes collected after growth for 1 or 7 days *in vitro* (logarithmic- and stationary-phases, respectively) were analyzed cytofluorimetrically. Cells were stained for exposure of enolase on the cell surface with an enolase-specific antiserum (EnoV; Rows **A** and **B**, right panels) and for membrane integrity with Propidium Iodide (PI; Rows **A** and **B**, center panels). Scatter (near- [FSC] and right-angle [SSC]) properties also were monitored (Rows **A** and **B**, left panels). The predominant scatter properties of logarithmic- and stationary-phase cells are denoted by the indicated polygons. Staining results for surface enolase exposure are presented for the entire logarithmic- and stationary-phase populations (gray histograms, Rows **A** and **B**, right panels) and separately for gated PI-negative (green histogram) and PI-positive (red histogram) stationary-phase cells (Row **B**, right panel overlay; “Events” are plotted to half scale). Quantification of cells within each population is indicated. Samples are from a single experiment, representative in pattern of five independent experiments. Compiled results from these independent determinations are presented in Row **C**; error bars represent the standard error of the mean (SEM) of these determinations. Comparable results were obtained from analyses performed with other enolase-specific antibody probes (see Supplementary Figure 1).

The change from logarithmic-phase growth to a stationary-phase culture of promastigotes is associated with a shift from larger (higher near-angle scatter [FSC] signal) parasites of low granularity (low right-angle scatter [SSC] signal) to smaller, more granular parasites (Figure 1, Rows A and B, left panels). These characteristics are consistent with previous characterizations of the differentiation of *Leishmania* parasites from procyclic to metacyclic promastigote forms (Bates and Tetley, 1993; Sacks, 1989; Saraiva et al., 2005; Williams et al., 2006). The virtually complete shift in population profiles indicates that *in vitro* metacyclogenesis under these conditions is efficient.

We evaluated parasite cell death in these *in vitro* cultures simply, assessing the loss of plasma membrane integrity by the uptake of propidium iodide (PI). PI-positive dead parasites arose spontaneously and abundantly among stationary-phase and not logarithmic-phase cultures (Figure 1, Rows A and B, center panels). We have observed significant variability in the extent of cell death among independent stationary-phase cultures (compare center panels of Figure 1 and Supplementary Figure 1, for example).

We found that enolase is exposed substantially on the surface of a large fraction of stationary-phase parasites (38.7%) and not on logarithmic-phase cells (3.5%; Figure 1, Rows A and B, right panels, gray histograms). Distinct, higher intensity staining is evident with PI-positive dead cells (Figure 1, Row B, right panel, red histogram).

Focusing on the stationary-phase parasites (Figure 1, Row B, center and right panels) in detail, the fraction of EnoV-positive cells (88.5%) and the EnoV-specific Mean Fluorescence Intensity (MFI = 9.1 × 10^0^) of the entire PI-positive subpopulation (marked in red) is substantially greater than the EnoV-positive fraction (21.8%) and the EnoV-specific MFI (5.1 × 10^0^) of the whole PI-negative subpopulation (marked in green). Note that, while the MFI’s of the EnoV-positive fractions of both PI-positive and PI-negative subpopulations (1.1 × 10^1^ and 1.0 × 10^1^, respectively) are similar, the staining profiles of those subpopulations are quite distinct.

**FIGURE 2:**
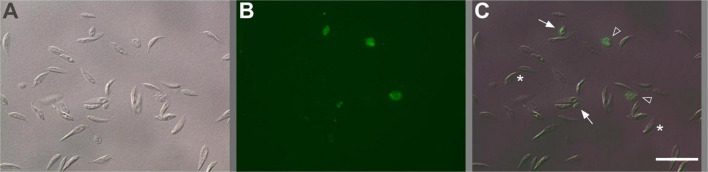
Microscopic visualization of cell surface enolase on *L. amazonensis* promastigotes. Promastigotes were analyzed microscopically after staining with hyperimmune rabbit anti-enolase serum followed by secondary goat anti-rabbit IgG conjugated to Alexa Fluor 488. Images were collected by panel **(A)** Differential Interference Contrast, and **(B)** fluorescence (Ex_λ_ = 470 ± 40 nm, Em_λ_ = 525 ± 50 nm) microscopy, and **(C)** merged. Solid arrows point to surface enolase-positive metacyclic promastigotes. Open arrowheads mark surface enolase-positive dead cells. Asterisks identify surface enolase-negative procyclic parasites. Bars represent 10 μm. Images are from a single experiment representative of two independent experiments.

Immunofluorescence imaging confirms that the surface display of enolase occurs on some small cells with characteristic metacyclic morphology (Figure 2, marked with solid arrows). Note that elongated procyclic parasites (Figure 2, exemplified with asterisks) do not display surface enolase. Other larger round enolase-positive cells (Figure 2, open arrowheads) likely are dead cells. The patchy peripheral staining of metacyclics contrasts with the well-distributed staining (also with granular foci) of dead cells.

We have observed this characteristic pattern of enolase externalization on the surface of viable metacyclic promastigotes consistently with distinct antibody probes, including antibodies directed to the N-terminal and C-terminal regions of human α-enolase (see Supplementary Figure 1). These observations identify the developmentally-regulated externalization of enolase, a molecule viewed classically as restricted to an intracellular locale, as another aspect of the complex developmental process of metacyclogenesis.

### Surface enolase of *L. amazonensis* does not have enzymatic activity

While enolase is externalized on a wide variety of organisms, enzymatic activity is not preserved consistently. We sought to assess whether *L. amazonensis* surface enolase retained enzymatic activity. Enolase activity was assessed as the fluoride-inhibitable conversion of 2-phosphoglycerate to phosphoenolpyruvate, as described previously (Ucker et al., 2012; see Section “Experimental Procedures”).

**FIGURE 3:**
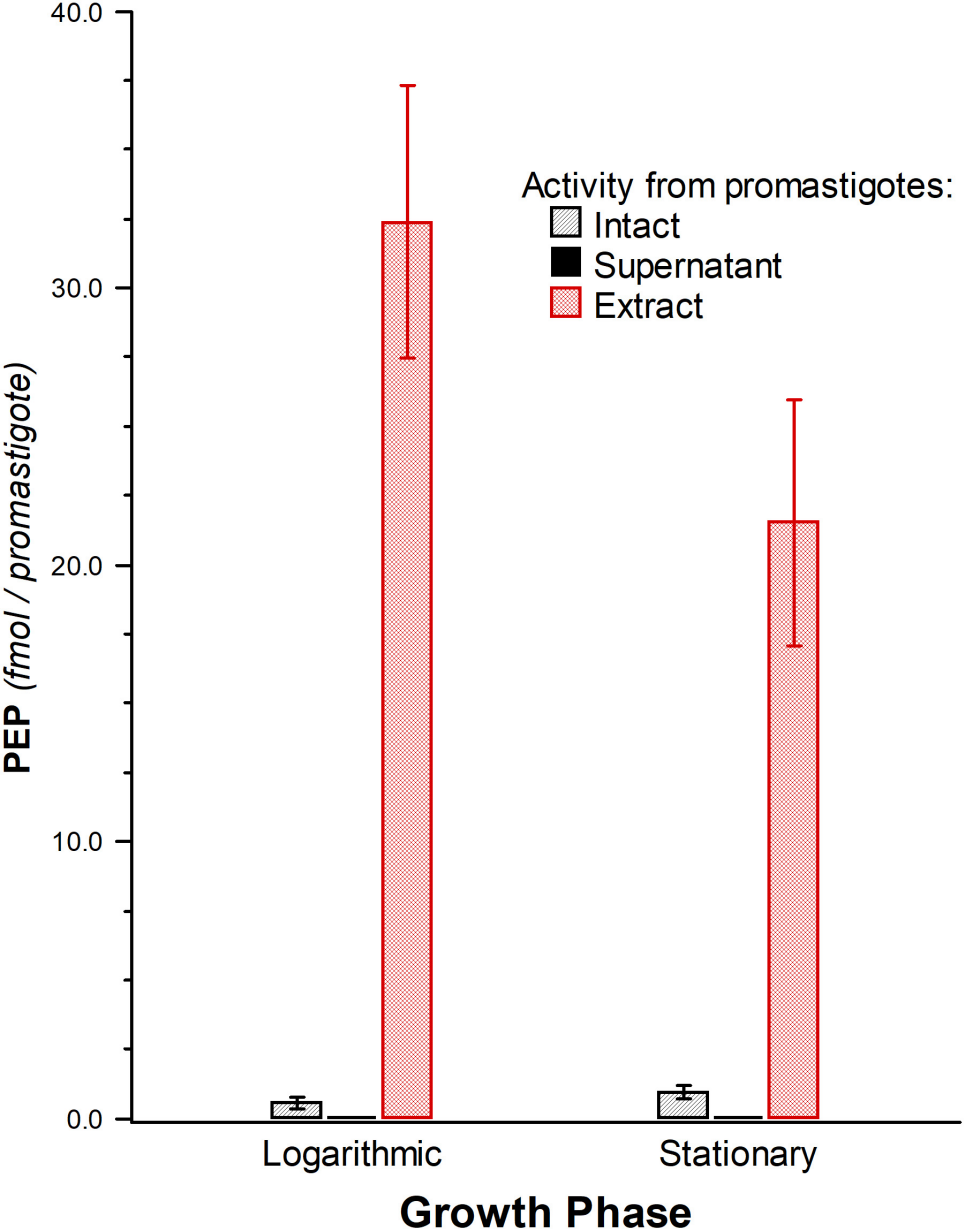
Analysis of the enzymatic activity of L. amazonensis promastigote enolase. Enolase activity was assessed as the fluoride-inhibitable conversion of 2-phosphoglycerate to phosphoenolpyruvate, as described in “Experimental Procedures.” Undisrupted logarithmic-or stationary-phase L. amazonensis promastigotes (5.0 × 10^7^/ml) or the sonicated extracts of cells at an equivalent density were incubated for 4 min at 25 °C (Intact 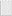 and Extract 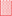, respectively). Reaction products were quantified spectrophotometrically. Activity in cell supernatants was assessed by incubating cells at an equivalent density in mock reactions without substrate, removing cells by centrifugation, and then incubating supernatants with substrate for an additional 4 min at 25 °C (Supernatant 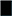). Error bars represent the standard error of the mean (SEM) of triplicate determinations.

Although a substantial fraction of stationary-phase promastigotes display enolase on the cell surface (Figure 1), enzymatic activity was detected exclusively in the extracts of those cells (Figure 3). Enolase activity was not detectable in suspensions of undisrupted stationary-phase cells nor in their supernatants. In fact, the minimal levels of enolase activities present in suspensions and supernatants of stationary-phase cells were indistinguishable from levels observed with surface enolase-negative logarithmic-phase cells (Figure 3). These data indicate that externalized *L. amazonensis* enolase lacks enzymatic activity.

### Surface enolase of *L. amazonensis* participates in promastigote attachment leading to infection of host macrophages

**FIGURE 4:**
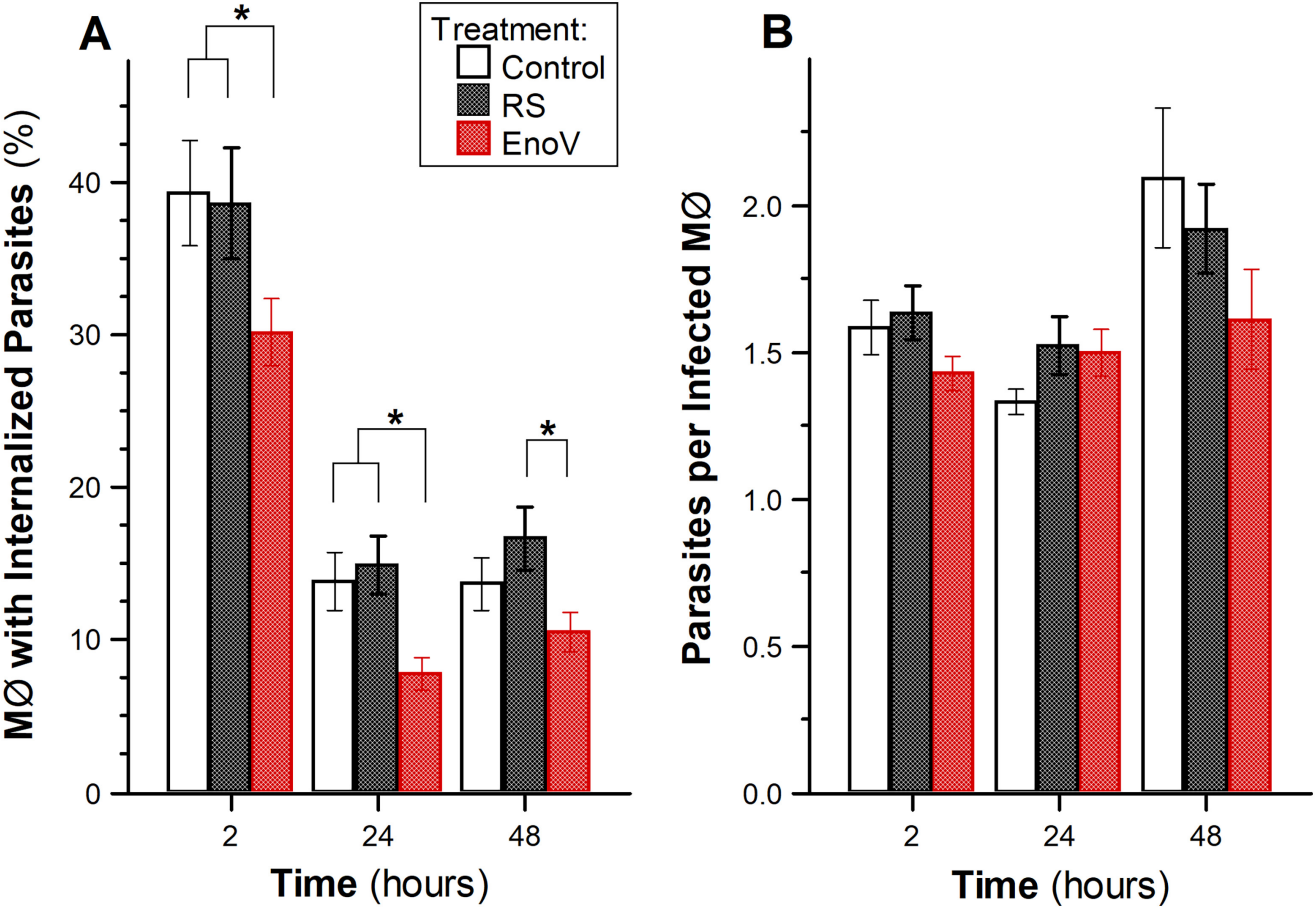
*Leishmania amazonensis* (*L. amazonensis*) surface enolase is an adhesion molecule involved in infection. Bone marrow-derived murine macrophages were incubated for 2 hr at 37 °C with promastigotes that had not been pretreated (Control 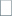); or that had been incubated previously with enolase-specific (EnoV; 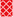) or preimmune (RS; 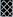) rabbit sera (MOI = 20 in all cases). Cells were washed after this infection period, and analyzed immediately or after further incubation at 37 °C for 24 or 48 hr. **(A)** Macrophages with internalized parasites were enumerated microscopically following washing after the indicated incubation. **(B)** The numbers of intracellular parasites per infected macrophage also were determined following incubation. Data are the compilation of 4 independent experiments (with at least 3 replicates per experiment). Error bars represent SEM of all determinations; statistically significant differences (*ρ ≤ 0.05) are indicated.

We examined the effect of blocking surface enolase on infection by *Leishmania amazonensis* promastigotes. Bone marrow-derived macrophages from C57BL/6 mice were infected with promastigotes that had been pre-treated with EnoV or control sera or that were not pretreated. Parasites are internalized rapidly by these cells (Courret et al., 2002). As shown in Figure 4A, pretreatment of promastigotes exclusively with the enolase-specific serum reduced the number of parasites internalized by macrophages. The diminution in the number of macrophages with internalized parasites over time affirms that not all parasites initially internalized establish productive infections.

We observed a very modest expansion in the number of parasites per infected macrophage by 48 hr post-infection (Figure 4B). By this time, all internalized parasites had differentiated morphologically into amastigotes. EnoV pretreatment exerted no statistically significant effect on this amastigote proliferation. In sum, the data of Figure 4 show that pretreatment of promastigotes with the enolase-specific serum reduces infectivity but does not affect significantly the proliferation of parasites within infected host cells.

**FIGURE 5:**
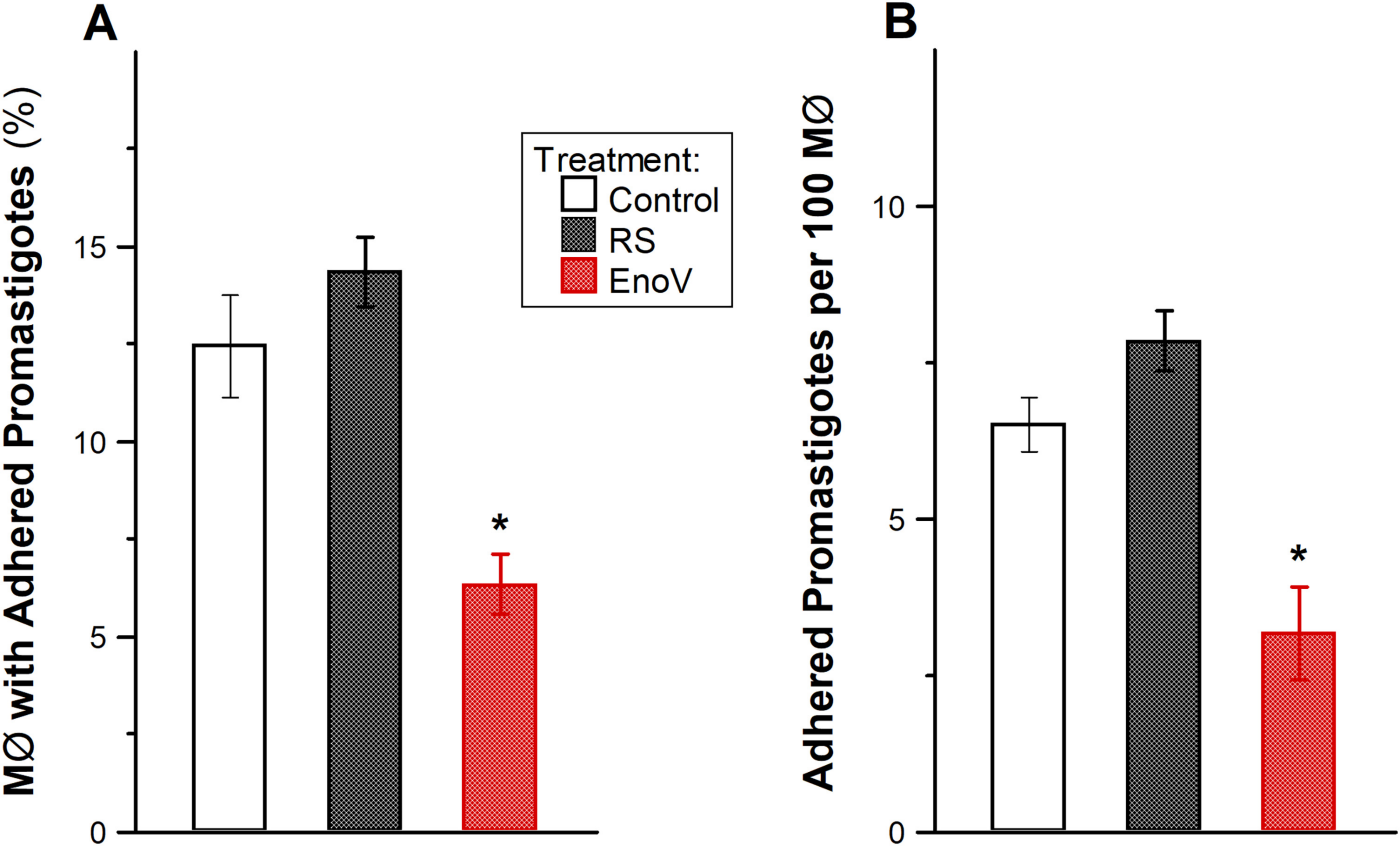
Surface enolase participates in adhesion of L. amazonensis promastigotes to host macrophages. Adhesion to bone marrow-derived murine macrophages of promastigotes that had not been pretreated (Control; 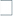) or that been incubated previously with enolase-specific (EnoV; 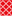) or preimmune (RS; 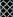) rabbit sera (MOI = 20) was monitored in the absence of phagocytic internalization. Internalization of infecting parasites was prevented by pretreatment with cytochalasin D (5 μM, 30 min) and parasite adhesion was assessed after 30 min of incubation at 37°C by microscopic enumeration following washing and fixation **(A)**. Alternatively, internalization was precluded by incubation at 4°C **(B)**. Macrophages and promastigotes were allowed to interact for 2 hr, at which time parasite adhesion was assessed as above. Data are the compilation of 3 independent experiments (with at least 3 replicates per experiment). Error bars represent SEM of all determinations; statistically significant differences (*ρ ≤ 0.05) are indicated.

In order to test this conclusion, we focused specifically on the role of surface-exposed enolase in the adhesion of promastigotes to host cells, dissociated from subsequent internalization. Phagocytosis was prevented either by incubation at 4 °C or by pretreating host macrophages with cytochalasin D (to inhibit dynamic actin-dependent processes). The results in both cases were similarly striking: we observed that blocking surface enolase on promastigotes with the EnoV serum specifically inhibited their adhesion to host macrophages by greater than 50%. The data in Figure 5A, from experiments with cytochalasin D, in which binding was allowed to ensue for only 30 min, reveal the significant inhibition of promastigote adhesion to macrophages exerted by blocking of surface enolase. Here, we examined the extent to which macrophages associated with promastigotes. We considered this issue from a different perspective in the low temperature experiments. As shown in Figure 5B, we tallied the numbers of promastigotes associating with macrophages and obtained a complementary result.

## Discussion

The externalization of enolase to the cell surface of *Leishmania amazonensis* is a striking, developmentally regulated event. While the surface display of enolase has been described previously in other *Leishmania* species, especially including the closely related New World species *L. mexicana*, the strict stage-specific regulation of enolase exposure has not previously been detailed. Work of Quiñones et al. (2007), in which *L. mexicana* parasites that express surface-exposed enolase have promastigote-like morphology, is consistent with this view.

Specific molecular interactions with host cells are fundamental for the establishment of *Leishmania* infections. Several virulence factors have been characterized that enable these interactions, some of them acquired or modified during metacyclogenesis. Among these are major surface protease (gp63), promastigote surface antigen 2 (gp46), and lipophosphoglycan (Beetham et al., 2003; McConville et al., 1992; Yao et al., 2003). Other bridging molecules, including activated complement proteins and antibodies, are derived from the host (Chu et al., 2010; Da Silva et al., 1989). Significantly, for most *Leishmania* species, blockade of any of these molecules leads only to partial inhibition of infection, underscoring the multifaceted, cooperative, and parallel pathways involved in facilitating infection. Our data demonstrating a role for surface enolase in the binding of promastigotes to murine macrophages identifies surface enolase as an additional virulence factor of *L. amazonensis*. Especially with regard to the developmental regulation of its surface expression and its cooperative role in adhesion, surface enolase manifests the characteristic behavior of a virulence factor. Other of its properties make enolase an unusual and especially intriguing virulence factor. How enolase functions as an adhesion factor in this context is the subject of ongoing work.

Enolase is known primarily as an intracellularly localized enzyme of central metabolic function. On the other hand, the externalization of enolase to the cell surface was first recognized in monocyte cell cultures because of the ability of exposed enolase to bind and serve as a receptor for plasminogen (Miles et al., 1991). Plasminogen binding was of modest affinity, with a circa 1 μM K_*d*_ (Redlitz et al., 1995). Surface enolase was later identified as a characteristic and abundant cell surface marker on apoptotic cells of all tissue types (Ucker et al., 2012). Apoptotic enolase externalization may account for the earlier attribution of enolase display to presumptive viable cells in proliferating cultures. Similarly, surface enolase was identified on tumor cells (Capello et al., 2011; Chang et al., 2006; Seweryn et al., 2009; Tu et al., 2010), where a role in promoting tumor metastasis via plasminogen activation and extracellular matrix degradation was postulated.

Independently, plasminogen binding to pathogenic bacteria led to the identification of cell surface enolase (Bergmann et al., 2003; Lottenberg et al., 1994; Pancholi and Fischetti, 1998). It was hypothesized – and subsequently demonstrated – that the activation of enolase-bound plasminogen facilitates fibrinolysis and pathogen dissemination (Attali et al., 2008; Hussain et al., 2022; Zhao et al., 2024). A wide variety of pathogenic bacteria (Candela et al., 2009; Lee et al., 2015; Toledo et al., 2012; Yavlovich et al., 2007) and other unicellular pathogens, from fungi to plasmodia (Dasari et al., 2019; Ghosh et al., 2011; Jong et al., 2003; Mundodi et al., 2008; Schreiner et al., 2012) also externalize enolase and bind plasminogen. This scenario extends to *Leishmania* species as well (Avilán et al., 2011; Swenerton et al., 2011; Vanegas et al., 2007). The significance of plasminogen binding for *L. mexicana* pathogenicity has been demonstrated by Avilán and co-workers, who documented the substantial resistance of plasminogen-deficient (*plg*^–/–^) mice to *L. mexicana* infection (Maldonado et al., 2006). Provocatively, comparison of *L. amazonensis* strains reveals a correlation between the abundance of membrane-associated enolase and virulence (Tano et al., 2022).

The binding of plasminogen to α-enolase is dependent on its lysine-rich carboxyl terminal region (Miles et al., 1991), which is maintained phylogenetically in the enolase genes of bacteria and other pathogens (Derbise et al., 2004; Nakayasu et al., 2017; Zhao et al., 2024). Other glycolytic enzymes that have been found to be exposed on the surface of apoptotic cells and pathogens, especially including glyceraldehyde-3-phosphate dehydrogenase [GAPDH; (Bednarek et al., 2024; Bergmann et al., 2004; Lama et al., 2009; Matsunaga et al., 2018; Ucker et al., 2012)], also incorporate a lysine-rich carboxyl terminal region to which plasminogen binds (Bergmann et al., 2004; Kumar et al., 2024). The seemingly parallel non-metabolic function of different glycolytic enzyme molecules has confounded the deconvolution of their individual contributions.

Uniquely in trypanosomatids such as *Leishmania* species, intracellular enolase does not associate with most of the other enzymes of the glycolytic pathway, which are segregated in glycosomes. Glycosomes are peroxisome-related organelles harboring the proximal elements of the glycolytic pathway; the distal three glycolytic enzymes, including enolase, are extra-glycosomal (Michels et al., 2006; Opperdoes, 1990; Parsons, 2004). Consistent with the lack of association of non-glycosomal and glycosomal enzymes, the externalization of enolase in *T. cruzi* (Avilán et al., 2011) appears to occur without the involvement of [glycosomal] triosephosphate isomerase (Cortés-Figueroa et al., 2008). In multiple experiments employing antibodies reactive with other glycolytic enzymes of *L. amazonensis*, we have seen no evidence of the externalization of glycosomally localized glycolytic enzymes, such as GAPDH (data not shown). This is a physiological context, then, in which to examine the non-metabolic role of enolase, dissociated from that of other glycolytic enzyme molecules.

We have not explored how enolase is transported to the cell surface of *L. amazonensis*. Work in yeast (Miura et al., 2012) suggests that an unconventional, SNARE-driven process could be involved. One proposed mechanism is that cell surface enolase arises by passive capture of soluble molecules secreted or released upon lysis (Jong et al., 2003; Mundodi et al., 2008). Evidence from studies with *L. donovani* (Swenerton et al., 2011) implicates the trypanosomatid protease oligopeptidase B (Motta et al., 2019) in the processing of surface enolase, excluding the “passive capture” model. In *L. amazonensis*, oligopeptidase B is expressed constitutively through all stages (de Matos Guedes et al., 2007), clarifying that it likely is not solely responsible for the developmentally regulated externalization of enolase.

The surface exposure of enolase at the infective stage in the *L. amazonensis* life cycle is reminiscent of the externalization of phosphatidylserine by metacyclic promastigotes (Wanderley et al., 2009). The exposure of phosphatidylserine during *Leishmania* metacyclogenesis itself parallels the externalization of phosphatidylserine during the process of metazoan apoptosis, an example of “apoptotic mimicry” (Barcinski et al., 2003; de Freitas Balanco et al., 2001; Wanderley et al., 2020). The externalization of the negatively charged phospholipid phosphatidylserine, normally cloistered in the inner leaflet of viable metazoan cells, is externalized specifically during apoptosis and plays an essential role in apoptotic cell clearance (Fadok et al., 1992; Hoffmann et al., 2001). On the surface of *L. amazonensis*, phosphatidyserine is a virulence factor that facilitates phagocytosis of infective parasites by target macrophages (França-Costa et al., 2012; Wanderley et al., 2006, 2013). In parallel, dead parasites expose phosphatidylserine that also contributes to virulence. While differentiating into the infective metacyclic form, both *in vitro* and in the sandfly vector, *L. amazonensis* promastigotes undergo a cell death process, apparently due to nutrient deprivation, that recapitulates metazoan apoptosis (Debrabant and Nakhasi, 2003; Serafim et al., 2012). Importantly, dead *L. amazonensis* promastigotes with phosphatidylserine exposed are engulfed and reduce the leishmanicidal capacity of macrophages (de Freitas Balanco et al., 2001), thus acting in trans to enhance the infectivity of viable *Leishmania* parasites (Wanderley et al., 2009).

Enolase externalization, too, appears to be an exemplar of apoptotic mimicry. Surface enolase of both apoptotic cells and *Leishmania* lacks enzymatic activity [this report and (Avilán et al., 2011; Quiñones et al., 2007; Swenerton et al., 2011; Ucker et al., 2012)]. On the surface of apoptotic cells, enolase may serve as an attachment molecule directing specific recognition. Apoptotic cells bind to and are recognized by virtually all cell types, notably including macrophages, and modulate the inflammatory responses of those cells (Birge and Ucker, 2008; Cvetanovic and Ucker, 2004; Cvetanovic et al., 2006; Fadok et al., 1998; Lucas et al., 2003; Voll et al., 1997). Exposed phosphatidylserine is not involved in this immune modulation (Gomes et al., 2022). Enolase was identified in a proteomic search for surface proteins linked to the triggering of apoptotic immunosuppression (Ucker et al., 2012). The host immune response to *L. amazonensis*, especially in its initial stages, also is characterized as immunologically quiescent, dependent in part on immunosuppressive IL-10 expression (Ji et al., 2003; also see Chu et al., 2010). On the surface of *Leishmania*, enolase may be important for inducing this suppressive state. Swenerton et al. (2011) found that oligopeptidase B-dependent activity, directed to surface enolase or other unidentified target(s), exerts a major impact in maintaining macrophage quiescence in response to infection.

The role of surface enolase as an immunoregulatory virulence factor of potential therapeutic value, and the link between that function and its plasminogen binding activity, remains to be elucidated. Another intriguing question is whether dead promastigotes, via their surface enolase, contribute to the induction of immune quiescence, just as they enhance *Leishmania* infectivity.

## Experimental procedures

### Mice

Female C57BL/6 and BALB/c mice were obtained from the Federal University of Rio de Janeiro Central Mouse Facility. All mice were maintained under specific conditions and used at 6–8 weeks of age according to the protocols approved by the Ethics Committee on the Use of Animals (CEUA - MAC047) of the Federal University of Rio de Janeiro.

### Parasites

Amastigotes of *L. amazonensis* (strain “Josefa” [WHOM/BR/75]) were recovered from lesions of infected BALB/c mice. Amastigotes were subsequently transformed into promastigotes *in vitro* by culturing at 23 °C in Schneider’s *Drosophila* medium, pH 7.2 (Sigma), supplemented with fetal bovine serum (FBS, 10% v/v; Sigma), L-glutamine (4 mM), penicillin (100 U/ml), and streptomycin (100 μg/ml). Promastigotes were passaged every 3 days and maintained for a maximum of six sequential passages.

### Bone marrow derived macrophages

Bone marrow derived macrophages were generated from C57BL/6 mice at 8 weeks of age. Fresh bone marrow was cultured (37 °C, 5% CO_2_) in Dulbecco’s Modified Eagle’s Medium (Gibco) supplemented with FBS (10%; Gibco) and recombinant macrophage-colony stimulating factor (M-CSF, 20 ng/ml; PeproTech). Medium was refreshed after 5 d, and macrophages were harvested 5 d later.

### Macrophage infection and analysis

Macrophages were plated on glass coverslips (13 mm^2^) in wells of a 24-well plate (5 × 10^4^ cells/well; 37 °C, 5% CO_2_) and allowed to attach overnight. Promastigotes, either untreated or treated previously with EnoV or normal rabbit serum (Invitrogen; #10510), were added to macrophages in fresh medium at an MOI of 1:20. The mixed population was incubated at 37 °C or at 4 °C as indicated, prior to the determination of infection or adhesion. In the case of cytochalasin D inhibition of internalization, macrophages were treated with cytochalasin D (5 μM), or left untreated, for 30 min and then washed with PBS (Sigma) prior to the addition of parasites.

Parasite burden was quantified by optical microscopy (100× oil immersion objective) following additional washing in PBS, fixation in methanol (Dinâmica), and hematoxylin and eosin staining (Renylab, MG, Brazil; #80002670086).

### Antibody staining and analysis

Promastigotes were washed twice in PBS, resuspended (1.0 × 10^6^/ml) in PBS Block (PBS plus bovine serum albumin [0.5%; Sigma] and normal mouse serum from C57BL/6 mice [2%]), and incubated on ice for 30 min. After washing in PBS, they were incubated with an enolase-specific antiserum, EnoV [*Leishmania mexicana* enolase-specific hyperimmune rabbit serum, 1:50 dilution in PBS; (Quiñones et al., 2007)] and incubated on ice for 30 min. Parasites then were washed twice in PBS and incubated with a goat anti-rabbit IgG secondary antibody for 30 min on ice. For cytofluorimetric analysis, the secondary antibody was conjugated to phycoerythrin (PE; Abcam ab97070, 1:1000 dilution). For fluorescence microscopy, the secondary antibody was conjugated to Alexa Fluor 488 (Invitrogen A11008, 1:200 dilution). Parasites were washed twice more in PBS before analysis. Other enolase-specific reagents were used in some experiments, including a mouse monoclonal antibody specific for the N-terminal region of human α-enolase (sc271384; Santa Cruz Biotechnology; referred to as EnoD) and a polyclonal goat antiserum directed to a C-terminal region peptide from human α-enolase (sc-7455; Santa Cruz).

For cytofluorimetry, Propidium Iodide (PI, 2 μg/ml; Molecular Probes) was added to all samples immediately before analysis for the assessment of parasite viability. PE and PI fluors were excited simultaneously with a 488 nm laser; fluorescence signals were distinguished using a 575 ± 26 nm band-pass filter for PE and a 610 ± 20 nm band-pass filter for PI. Single-stained controls for PE and PI, as well as unstained cells, were used to calculate spectral overlap and compensation. Data were collected in a FACSCalibur machine (50,000 gated events per sample). For fluorescence microscopy, samples were resuspended in 150 μl of 4% formaldehyde in 0.1 M phosphate, pH 7.2, and adhered to slides previously coated with poly-L-lysine (0.01%). After fixation on slides, a drop of ProLong Gold (Invitrogen) was added, and cells were observed using specific Axioplan fluorescence optics (Zeiss MrC5; Ex_λ_ = 470 ± 40 nm, Em_λ_ = 525 ± 50 nm) and Axiovision v4.6 software. Cells also were visualized with Nomarski Differential Interference Contrast optics (Zeiss #333389).

### Enolase enzymatic activity assay

Enolase activity was assessed as the fluoride-inhibitable conversion of 2-phosphoglycerate to phosphoenolpyruvate. Undisrupted (“Intact”) logarithmic-or stationary-phase *L. amazonensis* promastigotes (5.0 × 10^7^/ml in enolase buffer [10 mM MgCl_2_ in PBS]) or the freeze/thaw extracts of those cells at an equivalent density (“Extract”) were incubated in 200 μl reactions. Reactions were started with the addition of 2-phosphoglycerate (to a final concentration of 3 mM). After 4 min of incubation at 25 °C, reactions were terminated by the addition of 800 μl of enolase stop buffer (10 mM MgCl_2_ and 3 mM NaF in PBS). Activity in cell supernatants was assessed by incubating cells at an equivalent density in mock reactions without substrate, removing cells by centrifugation (13,000 rpm for 5 min. in a table-top microfuge), and then incubating supernatants with substrate for an additional 4 min at 25 °C. Reactions were quantified spectrophotometrically (λ = 240 nm). The molar extinction coefficient of phosphoenolpyruvate at 240 nm is 1.44 × 10^3^.

### Statistical analysis

Data were tested for normal distributions, analyzed by one-way ANOVA using GraphPad PRISM software (version 9.0. 2020), and subjected to post-test using Tukey’s Multiple Comparison Test method at a 95% confidence level.

## Data Availability

The raw data supporting the conclusions of this article will be made available by the authors, without undue reservation.
