# Supplementary material for: Surface enolase is a virulence factor of Leishmania amazonensis metacyclic promastigotes involved in adhesion to macrophages
Source: Front Microbiol. 2026 Jun 10;17:1789827. doi: 10.3389/fmicb.2026.1789827 (PMC13291145; doi:10.3389/fmicb.2026.1789827)
Supplement: Supplementary file 1 [file Image_1.pdf]

Supplementary Figure 1.

L. amazonensis **surface exposure of enolase - detection with an antibody directed to human α-enolase.**

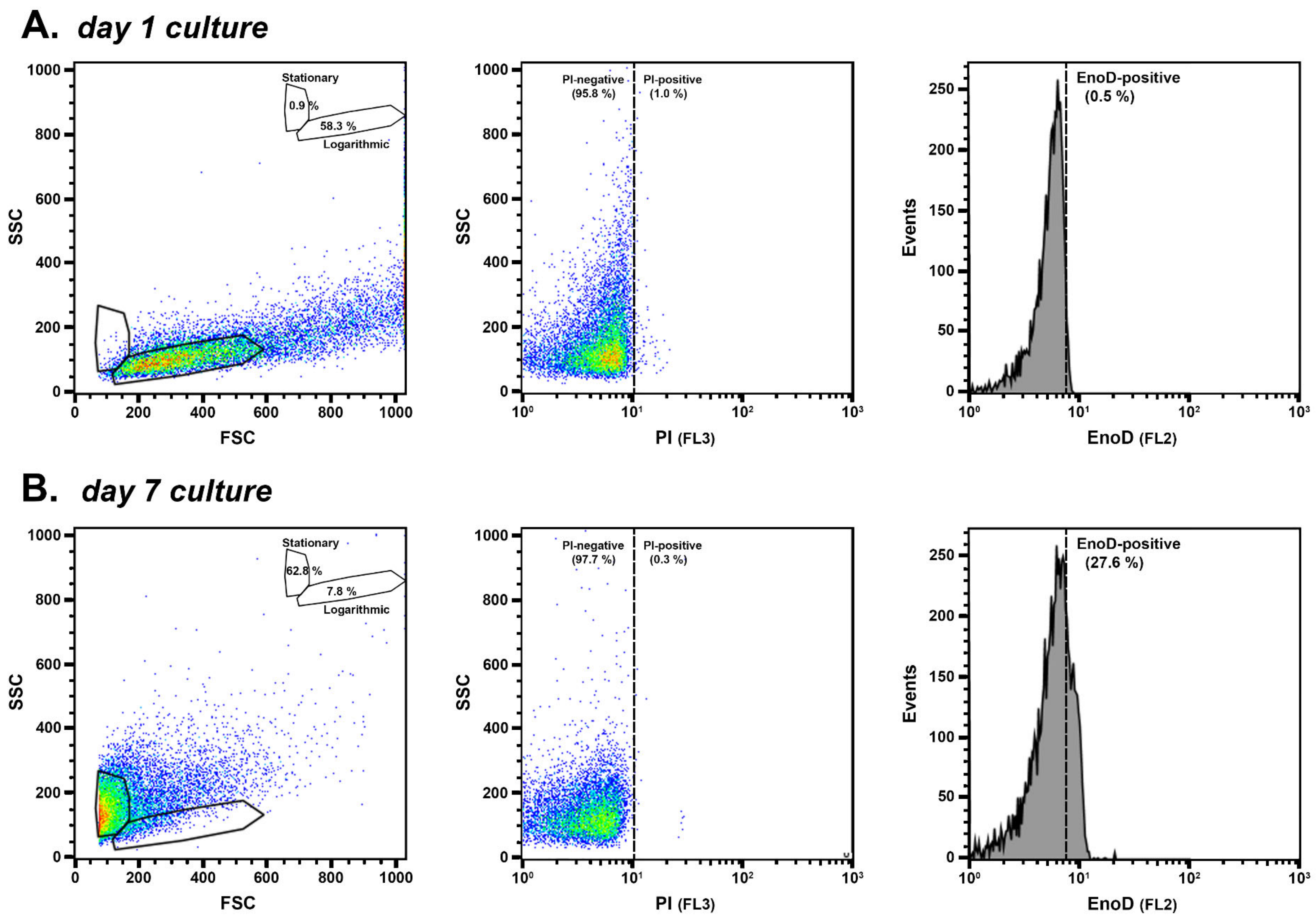

L. amazonensis promastigotes collected after growth for 1 or 7 days *in vitro* (logarithmic- and stationary-phases, respectively) were analyzed cytofluorimetrically, as in **Figure 1**, but with a mouse monoclonal antibody specific for the N-terminal region of human α-enolase. Cells were stained for exposure of enolase on the cell surface with the anti-human α-enolase antibody, conjugated to Alexa Fluor 488 (EnoD; Rows **A** and **B**, right panels) and for membrane integrity with Propidium Iodide (PI; Rows **A** and **B**, center panels). Scatter (near- [FSC] and right-angle [SSC]) properties were monitored (Rows **A** and **B**, left panels). As in **Figure 1**, the predominant scatter properties of logarithmic- and stationary-phase cells are denoted by the indicated polygons. Staining results for surface enolase exposure are presented for the entire logarithmic- and stationary-phase populations (gray histograms, Rows **A** and **B**, right panels), ungated with respect to PI (due to the infrequency of PI-positive cells in this experiment). Quantification of cells within each population is indicated. Samples are from a single experiment.
